# A Comparison of Three Methods to Measure Asthma in Epidemiologic Studies: Results from the Danish National Birth Cohort

**DOI:** 10.1371/journal.pone.0036328

**Published:** 2012-05-11

**Authors:** Susanne Hansen, Marin Strøm, Ekaterina Maslova, Erik Lykke Mortensen, Charlotta Granström, Sjurdur F. Olsen

**Affiliations:** 1 Centre for Fetal Programming, Department of Epidemiology Research, Statens Serum Institut, Copenhagen, Denmark; 2 Department of Epidemiology, Harvard School of Public Health, Boston, Massachusetts, United States of America; 3 Department of Nutrition, Harvard School of Public Health, Boston, Massachusetts, United States of America; 4 Institute of Public Health and Center for Healthy Aging, University of Copenhagen, Copenhagen, Denmark; Hospital for Sick Children, Canada

## Abstract

Asthma is a heterogeneous outcome and how the condition should be measured to best capture clinically relevant disease in epidemiologic studies remains unclear. We compared three methods of measuring asthma in the Danish National Birth Cohort (n>50.000). When the children were 7 years old, the prevalence of asthma was estimated from a self-administered questionnaire using parental report of doctor diagnoses, ICD-10 diagnoses from a population-based hospitalization registry, and data on anti-asthmatic medication from a population-based prescription registry. We assessed the agreement between the methods using kappa statistics. Highest prevalence of asthma was found using the prescription registry (32.2%) followed by the self-report (12.0%) and the hospitalization registry (6.6%). We found a substantial non-overlap between the methods (kappa = 0.21–0.38). When all three methods were combined the asthma prevalence was 3.6%. In conclusion, self-reported asthma, ICD-10 diagnoses from a hospitalization registry and data on anti-asthmatic medication use from a prescription registry lead to different prevalences of asthma in the same cohort of children. The non-overlap between the methods may be due to different abilities of the methods to identify cases with different phenotypes, in which case they should be treated as separate outcomes in future aetiological studies.

## Introduction

Recent decades have shown a concerning increase in the prevalence of asthma in both developed and developing countries [1]–[3]. According to the WHO, asthma is now one of the most common chronic respiratory diseases worldwide and can lead to reduced life quality for the affected individual as well as increasing health care costs for society [4].

Asthma is a complex and poorly defined syndrome characterized by several phenotypes, possibly with different aetiologies [5], [6]. There is currently no “gold standard” for the measurement of asthma and how the condition should be defined and measured in epidemiologic studies remains unclear. Most previous studies have used self-administered questionnaires to define asthma [7]. Questionnaires are useful in epidemiologic studies because of low costs, permitting larger sample sizes compared to intensive and expensive data collection methods, including bronchial challenge tests and reversibility tests [7], [8]. However, the appropriateness of using self-reported data to measure asthma in aetiological studies has been debated, mainly due to problems associated with recall of events and individual differences in symptom perception [9]. In Scandinavia, there is also the possibility of using population-based register data to obtain information about asthma [10], [11]. These registries can be useful as they do not depend on recall and allow for complete follow-up.

This study aimed to determine the prevalence of asthma in a population of children using three classification methods, including self-report, population-based hospitalization data, and population-based prescription data in a large prospective birth cohort, and to determine the agreement between the methods.

## Methods

### Study Population and Design

The study is based on a follow-up of the Danish National Birth Cohort that enrolled more than 100,000 pregnancies from 1996–2003 and has been described in detail elsewhere [12], [13]. A comprehensive follow-up questionnaire that included standardized questions on asthma from a large worldwide collaboration study, the International Study of Asthma and Allergies in Childhood (ISAAC), was mailed to the mothers when the children were 7 years old. In total, 93,616 mother-child pairs were included in the follow-up and answers were obtained for 53,637 children (57%).

Mothers provided written informed consent on behalf of their children. The Regional Scientific Ethics Committee for the municipalities of Copenhagen and Frederiksberg approved all study protocols, and all procedures were in accordance with the Declaration of Helsinki.

### Measures of Asthma

Self-reported asthma was assessed from the follow-up questionnaire when the children were 7 years old. We defined a self-reported asthma case as a child with a positive answer to the question *“Has a doctor ever said that your child had asthma?”*. Current self-reported asthma was defined as a child with a self-reported doctor diagnosis of asthma plus a positive answer to the question *“Has your child had any wheezing symptoms within the past 12 months?”*.

We used the unique Danish Personal Identification Number (CPR number) to extract information about asthma from two population-based registers: the Danish National Patient Register (DNPR) and the Register of Medicinal Product Statistics (RMPS).

Information about hospital contacts was extracted from the DNPR, which includes mandatory information about all hospital admissions in Denmark collected since 1977, and since 1995, also emergency room and outpatient contacts. Diagnoses of asthma were based on the International Classification of Diagnosis system 10 (ICD-10). We defined a hospitalization case of asthma as a child who had an asthma diagnosis (J45.0, J45.1, J45.2, J45.8, J45.9, or J46.9) registered in the DNPR from birth to 7 years of age. Only the first registered asthma case was used.

Information about asthma medication use was extracted from the RMPS, which includes mandatory information about all prescriptions redeemed at Danish pharmacies since 1995. Medication use was based on the Anatomical Therapeutic Chemical Classification System (ATC). We extracted information using the following ATC codes: R03A, R03B, R03C, and R03D. We used a previously validated definition of asthma based on prescription data [14]. We defined a prescription case of asthma as a child who had redeemed any type of anti-asthmatic drug except for beta2-agonists as liquid, inhaled beta2-agonists only once or inhaled steroid only once from birth to 7 years of age. Similarly to the DNPR, only the first registered case of asthma was used.

### Statistical Methods

We calculated the 7-year prevalences of asthma by classification method and for the methods in combination. The prevalence according to the registries was calculated for the complete study population; that is 93,616 children, whereas the self-reported prevalence was calculated for those who had filled out the questions on asthma.

Asthma cases from the three classification methods were compared in two-by-two tables and the agreement among cases and non-cases for each method calculated. We looked at all possible combinations of the classification methods and therefore alternatively used each of the three methods as the comparison measure or ‘gold standard’. Cohen’s kappa coefficient was calculated to describe the overall agreement between the classification methods.

In supplementary analyses we modified the case definitions of asthma in order to ascertain possible phenotypes. Current self-reported asthma was compared to cases ascertained by the registries, excluding the first 3 years of age from the register data. We expected that the modified definitions would to a larger extent reflect current asthma and exclude transient or viral wheeze diagnosed as asthma in early childhood which later resolved.

All analyses were carried out in SAS statistical software (version 9.2; SAS Institute, Cary, North Carolina).

## Results

### Asthma Prevalence

**Figure 1:**
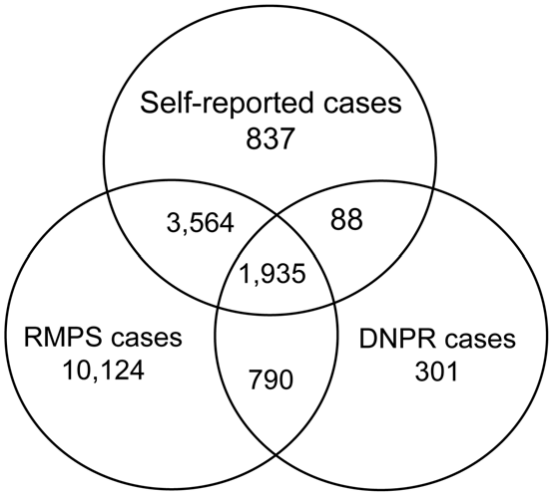
Comparison of asthma cases classified by self-report, the Danish National Patient Register (DNPR) and the Register of Medicinal Product Statistics (RMPS) in 53,637 children.

The 7-year prevalence of asthma for each classification method is shown in **Table 1**. The prevalence of self-reported asthma was 12.0%; of those 11.7% were currently experiencing wheezing symptoms. According to the hospitalization registry the asthma prevalence was 6.6%. The prescription registry yielded a prevalence of 32.7%. We found a substantial non-overlap between the cases ascertained by the three classification methods. Only 3.6% of the children were classified as cases during the first 7 years of age when all three classification methods were combined (**Figure 1**).

**Table 1:** 7-year prevalence of asthma according to self-report, the Danish National Patient Register and the Register of Medicinal Product Statistics.

Classification method	N cases/N non-cases	Prevalence %
Self-reports		
‘Self-reported asthma’: diagnosis of asthma given by a doctor (n = 53,637)	6,424/47,213	12.0%
‘Self-reported current asthma’: doctor diagnosis + current wheezing (n = 19,146)	2,244/16,902	11.7%
Danish National Patient Register		
Hospital cases (n = 93,616)	5,861/87,755	6.6%
Register of Medicinal Product Statistics		
Prescription cases (n = 93,616)	30,099/63,517	32.2%
Combination of methods		
Self-reported asthma + hospital cases + prescription cases (n = 53,637)	1,935/51,207	3.6%

### Classification Agreement

The overall agreement between self-reported asthma cases compared with cases ascertained by the hospitalization registry (k = 0.38) and the prescription registry (k = 0.37) was similar **(** **Table** **2** **)**. The overall agreement between cases ascertained by the two registries was lower (k = 0.21).

**Table 2:** Conditional distributions of asthma cases and non-cases classified by self-report, the Danish National Patient Register (DNPR) and the Registry of Medicinal Product Statistics (RMPS).

	Yes/Yes	No/No	Yes/No	No/Yes	Kappa
Self-report/DNPR	2,023	46,122	4,401	1,091	0.38
RMPS/Self-report	5,499	36,209	925	11,004	0.37
DNPR/RMPS	5,211	62,867	650	24,888	0.21

Among cases identified in the hospitalization registry and the prescription registry, the proportion of children who also had a self-reported doctor diagnosis of asthma was 65% and 33%, respectively (**Table 3**). If the self-reported cases were used as the reference, the proportions of cases also classified by the hospitalization registry and the prescription registry were 31% and 86%, respectively. 17% of the prescription cases were also hospital cases, whereas 90% of hospital cases were classified as cases by the prescription registry. In general, the three methods showed better agreement with regard to classifying non-cases with the proportions of agreement ranging from 72–99%.

**Table 3:** Agreement of asthma cases and non-cases classified by self-report, the Danish National Patient Register (DNPR) and the Register of Medicinal Product Statistics (RMPS).

	Agreement among cases	Agreement among non-cases
Self-report vs. DNPR	65%	91%
DNPR vs. Self-report	31%	98%
Self-report vs. RMPS	33%	98%
RMPS vs. Self-report	86%	77%
DNPR vs. RMPS	17%	99%
RMPS vs. DNPR	90%	72%

### Supplementary Analyses

**Table 4** shows the results from the supplementary analyses where registry information on the first 3 years of life was excluded. This modification of the case definitions did not change the overall agreements between the methods. Highest overall agreement was found between current self-reported asthma and the prescription registry (kappa = 0.35), followed by the agreement between current self-reported asthma and the hospitalization registry (kappa = 0.29), whereas the lowest agreement was found between the two registries (kappa = 0.21). The agreement between cases was lower for all comparisons compared to the measures that included the first 3 years of life (15–57% vs. 27–90%) whereas the agreement between non-cases remained high (93–99% vs. 72–99%).

**Table 4:** Agreement of asthma cases and non-cases classified by self-report (current asthma), the Danish National Patient Register (DNPR) and the Register of Medicinal Product Statistics (RMPS), excluding the first three years of age from the register data.

	Agreement among cases	Agreement among non-cases	Kappa
Self-report/DNPR	57%	91%	0.29
DNPR/Self-report	25%	98%	0.29
Self-report/RMPS	38%	93%	0.35
RMPS/Self-report	51%	89%	0.35
RMPS/DNPR	57%	93%	0.21
DNPR/RMPS	15%	99%	0.21

## Discussion

To our knowledge, this is the largest population-based study to simultaneously employ and compare three different asthma classification methods that are commonly used separately in epidemiological studies. Case ascertainment by means of self-report, a hospitalization registry, and a prescription registry yielded variable estimates of asthma prevalence during the first 7 years of age ranging from 6.6%–32.7%. Moreover, our study revealed a substantial non-overlap between cases identified by the three methods resulting in a prevalence of 3.6% if all three methods were combined to define asthma. Measures of agreement were low (kappa = 0.21–0.38).

Prevalence measures of asthma are difficult to compare across settings because of the lack of a “gold standard”. Another Danish study that employed ICD-10 codes of asthma in the DNPR found a prevalence of 5.0% of asthma in a cohort of children followed from birth to 12 years of age [15], which is consistent with our findings, despite an older study population. A population-based study from Norway that used data on prescriptions on anti-asthmatic medication from the Norwegian Prescription Database found a 12-month asthma prevalence of 9.1% among all Norwegian children aged 0–19 [16]. Compared to the prescription data prevalence reported in the present study, the Norwegian prevalence seems low, but this may be explained by the quite different outcome definitions and prevalence measures that were employed in the two studies. In a population of Canadian children aged 5 to 9 years, Yang et al. found a prevalence of asthma by self-reports of 15.7% and 21.1% using health claims [17]. The same question from the ISAAC questionnaire was used to define self-reported asthma as in our study. The health claims diagnosis was defined as at least one hospitalization for asthma at any time during the child’s life or two separate ambulatory or emergency room visits for asthma within a two year frame and had been validated against an expert consensus diagnosis of asthma [18]. While the self-report prevalence concurs quite well with our findings, the prevalence of the asthma health claims diagnosis cannot be directly compared to any of our results, since our registry data do not include primary care physicians’ diagnoses, whereas the employed Canadian health claims data do [18].

Overall, studies using self-report of asthma have yielded a range of different prevalences, illustrated for example by the ISAAC study, which reported 12-months prevalences of asthma ranging from 1.6–36.8% using the same standardized questionnaire in different populations [19].

The overall agreement of the asthma classification methods in our study was low, with kappa-values below 0.40 for all comparisons. This is in contrast to the results reported in the aforementioned study by Yang et al. that compared self-reported asthma with asthma diagnosis based on health claims data and found an overall agreement of kappa = 0.60 [17]. The better agreement with self-report in the study by Yang et al. may be due to the inclusion of primary care physicians’ diagnoses which are lacking in our study. However, as in our study, the agreement between the methods was higher when classifying non-cases.

A recent study by Nwaru et al. compared self-reported measures of asthma from a Finnish version of the ISAAC questionnaire with purchase of at least one anti-asthmatic medication during the preceding 12 months and found that the self-reported measures had a 98% agreement both with regard to classifying cases and non-cases of asthma [20]. Self-reported asthma was defined as a child with any wheezing symptom or use of asthma medication during the preceding 12 months plus ever asthma (with or without a doctor confirmation) and the definition of asthma based on the prescription registry included children with at least one prescription. The definitions therefore differ from those used in our study, both with regard to the prevalence period as well as the symptom-based definition which may explain the higher agreement of the compared measures in the Finnish study. Other studies that have compared self-reported measures of asthma and prescription data have found lower agreement between the methods [21], [22].

The variable prevalence estimates across the three methods that we employed, and the substantial non-overlap between the cases they identified, are noteworthy. The correct interpretation of this is hampered by the fact that we have not assessed the true prevalence of asthma in our population which would require a clinical assessment of each individual. It is reasonable to assume that the three methods we employed may have different error sources and thus variable degrees of misclassification for a given biological phenotype. However, for the case definition using the prescription register data, we used a validated definition of asthma that has previously been shown in a study by Moth *et al*. to have a specificity of 0.86 and a sensitivity of 0.63 compared with discharge information and a questionnaire completed by general practitioners in those without a discharge diagnosis [14]. For self-reported measures, poor recall of past events could have affected the results, although we believe this would not lead to any systematic over- or underreporting of the conditions.

While different error sources may explain some of the variance in the prevalences of asthma among the assessment methods, it is also possible that the three methods identify asthma cases with biologically distinct phenotypes. For example, the hospitalization registry may capture more severe phenotypes than the prescription registry or maternal self-report. The overrepresentation of more severe cases when using this classification method may however be appropriate for studies of aetiological associations if hospitalized cases have a different phenotypical aetiology compared to the cases identified using the other methods. In contrast, the prescription registry may identify a heterogeneous mix of cases, ranging from suspected asthma cases that may have been prescribed anti-asthmatic medication to clarify a diagnosis to more severe cases that continuously require anti-asthmatic treatment. A study showed that 13.9% of all children in Denmark aged 0–15 years had redeemed prescriptions of at least one anti-asthmatic drug in one year but not all of these children had received an asthma diagnosis leading to an overestimation of the asthma prevalence [23]. This is also true for the self-reported diagnosis for asthma that also may comprise a broad spectrum of cases with respiratory symptoms ranging from mild to severe, including wheezing.

Wheezing symptoms in young children aged 0–3 years are common and many are prescribed anti-asthmatic mediations [24]. These wheezing symptoms are often transient or viral-induced and later remit [5]. Including the transient wheezers in the asthma measures may dilute possible associations between exposures and clinically relevant asthma and this may have implications for studies of aetiological associations. When we excluded the first three years of life from the register data and compared it to current self-reported asthma we did not, however, find an overall increased agreement between the methods. This supports the conclusion that the three methods may not identify the same asthma cases and that the outcomes may represent different phenotypes of asthma.

The extent to which the non-overlap between the methods may be due to misclassification or, to different phenotypes being identified by each method, has implications for how to use these methods in studies where each individual has been assessed by more than one method simultaneously. Assuming that the methods identify a single phenotype, methods can be combined. In aetiologic studies of asthma where the aim is to estimate a relative risk for a given factor high specificity may be desirable even at the expense of lower sensitivity; this could be obtained by requiring that two or more methods agree on the asthma case ascertainment. For example, the self-reported measures could be used in combination with the prescription register definition to define an asthma case which in our study would lead to an asthma prevalence of 10.2% On the other hand, assuming that the methods identify different phenotypes we may need to do separate analyses, one for each case definition.

In conclusion, our large-scale prospective study comparing three different methods for asthma ascertainment revealed a substantial non-overlap between cases identified by the three methods. Reflective of either different biases or different phenotypical expressions of asthma, these classifications need to be carefully considered when deciding on asthma outcomes in future aetiological studies.
